# Prognostic Role of Tumor Microenvironment in DLBCL and Relation to Patients' Clinical Outcome: A Clinical and Immunohistochemical Study

**DOI:** 10.1155/2022/9993496

**Published:** 2022-01-17

**Authors:** Doaa Shams-Deen Ghorab, Ahmed Mohamed Helaly, Hoda Saleh El Mahdi, Moawiah Khatatbeh, Afaf Taha Ibrahiem

**Affiliations:** ^1^Pathology Department, Faculty of Medicine Mansoura University, Egypt; ^2^Basic Department, Faculty of Medicine Yarmouk University, Jordan; ^3^Forensic and Clinical Toxicology Department, Mansoura University, Egypt; ^4^Clinical Department, Faculty of Medicine Yarmouk University, Jordan

## Abstract

Diffuse large B cell lymphoma is the most common type of lymphoma in Egypt with an unfavorable prognosis. The tumor microenvironment is rich in immune response either T cells or macrophages. The current study is aimed at testing CD4, CD8, CD68, and MMP9 immunohistochemistry of DLBCL activities with the prognosis of the tumor. The results showed no positive relation between T cell and macrophage reaction to the tumor prognosis suggesting that this reaction is part of the tumor process and not a defense mechanism from the surrounding stroma.

## 1. Introduction

Diffuse large B cell lymphoma (DLBCL) is the most common subtype of non-Hodgkin lymphoma, accounting for 40% of new cases. Although DLBCL is recognized as a single entity by the World Health Organization, DLBCL is clinically and biologically heterogeneous and aggressive and includes several subtypes [1].

It can be cured in 60–70% of cases after first-line immunochemotherapy. Nevertheless, 30–40% of cases will experience recurrence or refractory disease after the initial response, which will dramatically reduce their survival. Several studies focused on the identification of new individual prognostic and risk stratification biomarkers and new potential therapeutic targets [2].

The interest regarding the importance of the microenvironment including adaptive immune response and macrophages in cancer is growing, and avoidance of immune control of tumor growth and spread is a hallmark of cancer [3, 4].

T cell-mediated immunity plays an important role in enhancing antitumor response. CD4+ and CD8+ are the two main lineages of T cells [5, 6]. Early data suggested that brisk infiltration of T cells in primary melanoma lesions was a positive prognostic factor [7]. Later, similar data has been found in other cancers including ovarian cancer [8], renal cell carcinoma (RCC) [9], bladder cancer [10], colorectal cancer (CRC), and also other solid cancers [11].

The expression of T cell-associated antigens is not seen in benign lymphoid proliferations and is uncommon in B cell non-Hodgkin lymphoma (B-NHL), most frequently occurring in the setting of chronic lymphocytic leukemia/small lymphocytic lymphoma [12]. Although CD4/CD8 respective proportions and function were reported as predictors of patient outcomes in cancer, studies were often unclear or contradictory in lymphomas [13, 14].

The role of tumor-associated macrophages (TAMs) also has been widely studied in the pathogenesis of various cancers, especially because of their controversial role. On the one side, they can kill tumor cells, but on the other side, they may favor tumor growth, invasion, and progression by inducing immunosuppression and synthesis of higher levels of angiogenic factors such as VEGF, interleukin 8 (IL-8), TNF-alpha, metalloproteases, and fibroblast growth factor 1 (FGF-1) [5].

Tumor-associated macrophages (TAMs) have recently been reported as an important factor in tumor growth and the progression of cancer. Previous studies confirmed that TAMs are associated with cancer survival in several organs such as hepatoma, gastric cancer, and lung cancer [15, 16].

The presence of MPs in a tumor can be indicative of several characteristics of a lymphoma's clinical signature, including prognosis as well as the efficacy of chemotherapy [17]. Even before cells become cancerous, MPs can add to their surrounding inflammatory environment, producing mutagenic substances like reactive oxygen species that may support or augment oncogenesis [18].

In this study, we aim to assess the expression of CD4 and CD8 as T cell markers and macrophage markers (CD68 for M1, MMP9 for M2) in cases of DLBCL and correlate the expression to other clinicopathologic features and clinical outcomes.

## 2. Materials and Methods

A retrospective study was carried out on formalin-fixed paraffin-embedded tissues for 65 patients with diffuse large B cell lymphoma. All clinical and pathological data were retrieved from archives of pathology lab, Oncology Center of Mansoura University. The study will include the lymphoma cases that received the same protocol of treatment.

Sections were cut from paraffin-embedded tissue blocks at 4 *μ*m and deparaffinized with xylene, then rehydrated with graded alcohols. Endogenous peroxidase was blocked with 0.3% hydrogen peroxide for 5 min. Then, antigen retrieval was done with heat in citrate; pH 6.0 for CD4, CD8, and CD68 and EDTA for MMP9.

CD4 (clone 4B12, dilution 1 AQ4: 100; Leica Biosystems), CD8 (clone 1A5, dilution 1 : 30; Leica Biosystems), MMP9 (2C3) 1 mL: sc-21733 (mouse monoclone, Santa Cruz Biotechnology at 1 : 100), and CD68 (Kp1) 7 mL, predilute, cat. No. 134M-18: mouse monoclonal, cell marque.

Detection kits used were cytoscan HRP (cell marque, cat. NO.951D-20).

Slides were counterstained with hematoxylin then dehydrated with alcohol and xylene.

## 3. Immunohistochemical Evaluation

Two pathologists scored the slides independently. The number of CD4+, CD8+, CD68, and MMP9-positive cells was estimated by counting all positive cells in 5 high-power (400x objective) monitor fields (HPF) (0.029 mm^2^ each); the mean number was assessed then scored as shown in Table 1.

The total score was calculated by multiplying both intensity and percentage score (Table 1).

## 4. Results

**Figure 1:**
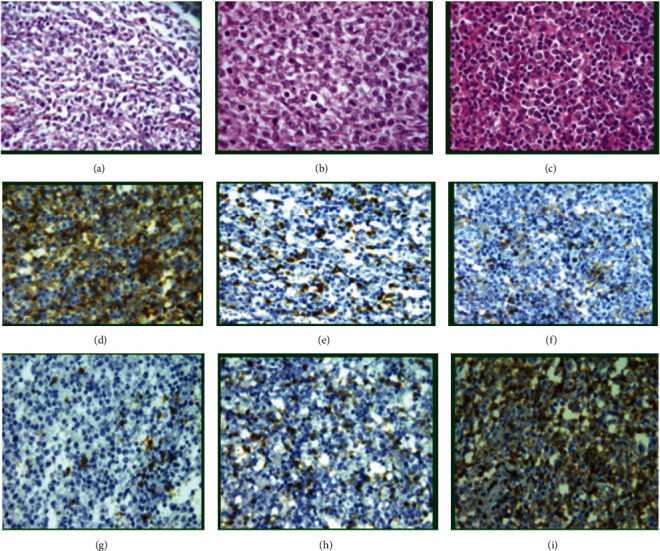
DLBCL with reactive appearing lymphocytes in the background (a–c, ×200); IHC staining for CD4 showed the variable intensity of CD4-positive T helper cells in the background of lymphoma cells (high, moderate, and low (d–f, respectively, ×200)). IHC for CD8 showed the variable intensity of CD8-positive cytotoxic T cells in the background of lymphoma cells (low, moderate, and high (g–i, respectively, ×200)).

Figure 1 shows reactive T lymphocytes in the background of DLBCL with H&E stain (a–c); reactive T helper cells are seen in the background of DLBCL as small brown stained cells with anti-CD4 monoclone with high, moderate, and low intensity (d–f, respectively). In addition, cytotoxic T cells are seen in the background of DLBCL as brown stained small cells with anti-CD8 monoclone with low, moderate, and high intensity (g–i, respectively).

**Figure 2:**
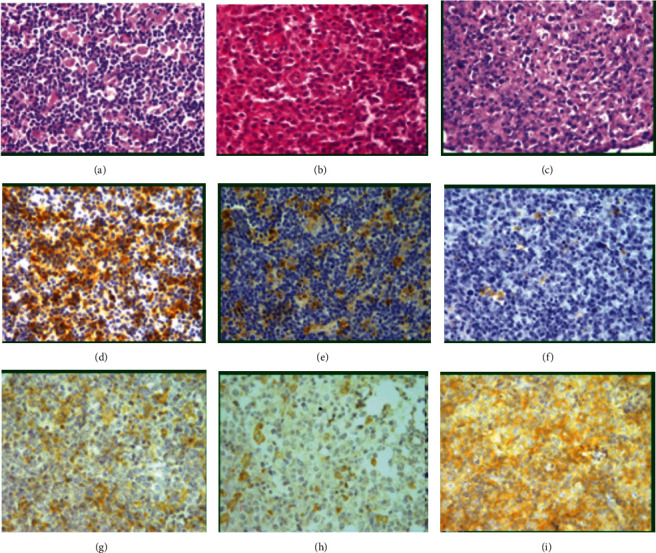
DLBCL with reactive appearing histocytes in the background (a–c, ×200); IHC staining for CD68 showed the variable intensity of CD68-positive histiocytic cells in the background of lymphoma cells (high, moderate, and low (d–f, respectively, ×200)). IHC for MMP9 showed the variable intensity of histocytes in the background of lymphoma cells (low, moderate, and high (g–i, respectively, ×200).

Figure 2 shows reactive histiocytes in the background of DLBCL with H&E stain seen as polygonal cells with abundant eosinophilic or foamy cytoplasm and rounded vesicular nuclei (a–c). Histiocytes or macrophage type M1 are seen as brown cells stained with CD68 with high, moderate, and low intensity (d–f, respectively). Reactive histiocyte (macrophages) type M2 are seen stained brown color with MMP9 with low, moderate, and high intensity (g–i, respectively).

The results compared the DLBC lymphoma cases with different prognostic markers. The tumor samples have been stained with CD4, CD8, CD68, and MMP9 immune stain markers. The stain markers were correlated with the following parameters: age, gender, bulky disease, B symptoms, anemia, LDH, performance status and extranodal involvement, bone marrow and CNS involvement, staging, and IPI scoring. The current research also focused on Ki67, BCL2 expression, relapse, overall survival, and therapy response. Each immune marker sample was categorized into 2 groups. The first one is the low expression group, and the second one is the high expression group. The 4 markers CD4, CD8, CD68, and MMP9 were compared to the different prognostic markers aimed at detecting any effect of these immune marker expressions on the prognosis of the tumor (Tables 2 and 3). The results showed that immune marker expression has no significant correlation with the prognosis of the different DBCL cases at any parameter. That is to say, whether the expression of the markers CD4, CD8, CD68, or MMP9 was high or low, there was no impact on the different prognostic indexes of the study.

## 5. Discussion

The current work tracked immunohistochemistry marker CD4, CD8, CD68, and MMP9 staining in diffuse large B cell lymphoma cases. The markers were evaluated in the conjunction with different possible prognostic parameters including age, gender, bulky disease, the presence of B symptoms, anemia, LDH level, performance status, extranodal involvement, bone marrow and CNS involvement, staging IPI scoring, and associated Ki67 and BCL2 expression. The study examined the possible relation between the immune panel and relapse of lymphoma cases, the overall survival, and therapy response. The current study showed no relation between the immune activity and any of the different prognostic markers of lymphoma. Both CD4 and CD8 expression represented the T activity, and CD68 and MMP9 reflected the role of the macrophage. It was hypnotized that both components are part of the host defense that will defend against the tumor, and it was expected that the higher the immune staining the better the prognosis. However, it is not the case here in this most common type of lymphoma. And here, the question could be raised about the role of the immune mechanism not related to the prognosis at all. It is suggested that such a response is a supporting or scavenger servant of the tumor. More detailed work is needed to track the role of both T cells and macrophages in favor of the tumor, not against it. Also, some authors could claim that both T cells and macrophages are unrelated to the tumor component as they are well-differentiated, but why these cells are active in the tumor microenvironment is another point of research. In this study, it can be said that the immune activity in the lymphoma microenvironment in this DLBCL Egyptian sample is not an adaptive response. Unlike the current outcome, experimental work tested the CD4/CD8 ratio in DLBCL samples and concluded that T cell activity may play a role in DBCL lymphoma prognosis [19]. It is proposed that lymphoma is a heterogeneous syndrome. Another relevant study showed no relation between CD4 and CD5 and the prognosis of DLBCL as in the current outcome; however, CD5 showed adverse relation to the patient prognosis [20]. These conclusions support the notion that immune activation in the tumor microenvironment is part of the tumor process. These findings have been tracked by a microarray gene study in poor prognosis DLBCL and mantle lymphomas. It was demonstrated that the CD5 poor prognosis parameter was associated with high expression of integrin beta1 and/or CD36 adhesion molecules [21].

As regarding the role of CD68 in the prognosis of DLBCL, the current research expressed no significant relation. Chinese research showed a positive impact of the monocyte activity demonstrated by CD68 and 163 immunohistochemistry in contrast to the current data [22]. Such variation puts the spot on the racial variation in the tumor process. However, another work supported the current data that CD68 lacks prognostic value about DLBCL outcome but supported the Chinese results that CD163 has a prognostic value. However, they admitted that the lymphocytes/macrophage ratio may have a better prognostic value [23].

MMP9 activity has been demonstrated in NHL suggesting aggressive behavior and poor outcome [24]. The current study examined the prognostic value of MMP1 in DLBC lymphomas to check if it reflects invasive behavior, yet MMP9 expressed no significant value. It seems that the interaction between stroma and the NHL lymphoma of current samples is weak unlike other tumors like breast cancer [25, 26]. Studies carried out on cervical squamous cell carcinoma showed that MMP1 is inversely related to CD4, CD8, and macrophage activity in the tumor microenvironment. The story is different in lymphoma cases [27]. Another study showed a negative relation between matrix metalloproteinase markers and the prognosis of lymphoma candidates [28]. The results regarding the prognostic role of macrophage markers were conflicting. A multicenter study concluded that macrophage markers indicated a good prognosis [29]. On the other hand, a meta-analysis work showed an unfortunate prognosis of DLBCL with macrophage immune staining. The current work expressed the 3^rd^ option of no relation with the disease outcome. This heterogeneous response suggests the heterogeneous nature of DLBCL and lymphoma in general. It is recommended to resub type DLBCL on a new molecular base [30].

## 6. Conclusion

The current study demonstrated no positive role of the panel of immunochemistry CD4, CD8, CD68, and MMP with the overall survival of the lymphoma prognosis. These markers suggest that immune reaction is not a defense response to the tumor because of no improvement of the prognosis, unlike other tumors. It is concluded that such a response may be a component of the tumor supporting the malignancy process.

## Figures and Tables

**Table 1:** Staining system of tumor cells for CD4, CD8, MMP9, and CD68.

The intensity of staining score		Percent of stained cells	
Score 0	No staining	Score 0	No. of cells
Score 1	Weak staining	Score 1	1-20% of cells were stained
Score 2	Moderate staining	Score 2	20-70% of cells were stained
Score 3	Strong staining	Score 3	70-100% of cells were stained
Total score 0-3 = low expression 4-9 = high expression			

**Table 2:** The relation between T lymphocytic prognostic markers CD4 and CD8 and the following parameters: age, gender, bulky diseases, B symptoms, anemia, LDH, performance status, extranodal involvement, bone marrow and CNS involvement, stage, IPI, Ki67, BCL2, relapse, overall survival, and therapy response.

	CD4		*P* value	CD8		*P* value
	Low Ex	High Ex		Low Ex	High Ex	
*Age*						
0-59	49.2%	13.8%	0.916	53.8%	9.2%	0.545
+=60	29.2%	7.7%		30.8%	6.2%	
*Gender*						
Male	40.0%	16.9%	0.065	49.2%	7.7%	0.631
Female	38.5%	4.6%		35.4%	7.7%	
*Bulky dis*						
-ve	63.1%	18.5%	0.494	66.2%	15.4%	0.109
+ve	15.4%	3.1%		18.5%	0.0%	
*B symptoms*						
-ve	53.8%	20.0%	0.062	60.0%	13.8%	0.196
+ve	24.6%	1.5%		24.6%	1.5%	
*Anemia*						
-ve	50.8%	15.4%	0.448	58.5%	7.7%	0.241
+ve	27.7%	6.2%		26.2%	7.7%	
*LDH*						
Normal	4.6%	1.5%	0.631	7.3%	0.0%	0.504
High	73.8%	20.0%		78.5%	15.4%	
*Performance status*						
1	61.5%	16.9%	0.864	64.6%	13.8%	0.615
2	15.4%	4.6%		18.5%	1.5%	
3	1.5%	0.0%		1.5%	0.0%	
*Extranodal involvement*						
0	60.0%	12.3%	0.235	61.5%	10.8%	0.569
1	15.4%	6.2%		16.9%	4.6%	
2	3.1%	3.1%		6.2%	0.0%	
*Bone marrow and CNS involvement*						
-ve	69.2%	20.0%	0.528	73.8%	15.4%	0.291
+ve	9.2%	1.5%		10.8%	0.0%	
*Stage*						
1	6.2%	4.6%	0.427	7.7%	3.1%	0.387
2	9.2%	1.5%		10.8%	0.0%	
3	40.0%	7.7%		38.5%	9.2%	
4	23.1%	7.7%		27.7%	3.1%	
*IPI*						
1-2	50.8%	10.8%	0.316	50.8%	10.8%	0.411
3-4	27.7%	10.8%		33.8%	4.6%	
*Ki67*						
Low exp.	15.4%	3.1%	0.494	13.8%	4.6%	0.267
High exp.	63.1%	18.5%		70.8%	10.8%	
*BCl2*						
Low exp.	26.2%	4.6%	0.306	24.6%	6.2%	0.365
High exp.	52.3%	16.9%		60.0%	9.2%	
*Relapse*						
No	55.4%	13.8%	0.645	56.9%	12.3%	0.387
1-12 m	9.2%	4.6%		13.8%	0.0%	
=+13 m	13.8%	3.1%		13.8%	3.1%	
*Overall survival*						
1-12 m	24.6%	6.2%	0.539	26.2%	4.6%	0.913
13-24 m	16.9%	7.7%		21.5%	3.1%	
=+25	36.9%	7.7%		36.9%	7.7%	
*Therapy response*						
Regressive	16.9%	6.2%	0.581	20.0%	3.1%	0.867
Stationary	21.5%	3.1%		21.5%	3.1%	
Progressive	40.0%	12.3%		43.1%	9.2%	

**Table 3:** The relation between macrophage marker CD68 and MMP9 expressions and the following parameters: age, gender, bulky diseases, B symptoms, anemia, LDH, performance status, extranodal involvement, bone marrow and CNS involvement, stage, IPI, Ki67, BCL2, relapse, overall survival, and therapy response.

	CD68		*P* value	MMP9		*P* value
	Low Ex	High Ex		Low Ex	High Ex	
*Age*						
0-59	26.2%	36.9%	0.321	26.2%	36.9%	0.753
+=60	20.0%	16.9%		13.8%	23.1%	
*Gender*						
Male	26.2%	30.8%	0.969	18.5%	38.5%	0.152
Female	20.0%	23.1%		21.5%	21.5%	
*Bulky dis*						
-ve	35.4%	46.2%	0.349	29.2%	52.3%	0.151
+ve	10.8%	7.7%		10.8%	7.7%	
*B symptoms*						
-ve	30.8%	43.1%	0.223	33.8%	40.0%	0.091
+ve	33.3%	20.0%		6.2%	20.0%	
*Anemia*						
-ve	29.2%	36.9%	0.656	24.6%	41.5%	0.521
+ve	16.9%	16.9%		15.4%	18.5%	
*LDH*						
Normal	3.1%	3.1%	0.633	1.5%	4.6%	0.472
High	43.1%	50.8%		38.5%	55.4%	
*Performance status*						
1	33.8%	44.6%	0.435	35.4%	43.1%	0.250
2	10.8%	9.2%		4.6%	15.4%	
3	1.5%	0.0%		0.0%	1.5%	
*Extranodal involvement*						
0	29.2%	43.1%	0.085	29.2%	43.1%	0.808
1	15.4%	6.2%		9.2%	12.3%	
2	1.5%	4.6%		1.5%	4.6%	
*Bone marrow and CNS involvement*						
-ve	38.5%	50.8%	0.155	35.4%	53.8%	0.587
+ve	7.7%	3.1%		4.6%	6.2%	
*Stage*						
1	6.2%	4.6%	0.870	6.2%	4.6%	0.463
2	4.6%	6.2%		6.2%	4.6%	
3	20.0%	27.7%		18.5%	29.2%	
4	15.4%	15.4%		9.2%	21.5%	
*IPI*						
1-2	26.2%	35.4%	0.455	27.7%	33.8%	0.298
3-4	20.0%	18.5%		12.3%	26.2%	
*Ki67*						
Low exp.	9.2%	9.2%	0.767	4.6%	13.8%	0.200
High exp.	36.9%	44.6%		35.4%	46.2%	
*BCl2*						
Low exp.	15.4%	15.4%	0.678	12.3%	18.5%	1.000
High exp.	30.8%	38.5%		27.7%	41.5%	
*Relapse*						
No	32.3%	36.9%	0.991	24.6%	44.6%	0.493
1-12 m	6.2%	7.7%		7.7%	6.2%	
=+13 m	7.7%	9.2%		7.7%	9.2%	
*Overall survival*						
1-12 m	16.9%	13.8%	0.627	13.8%	16.9%	0.690
13-24 m	10.8%	13.8%		7.7%	16.9%	
=+25	18.5%	26.2%		18.5%	26.2%	
*Therapy response*						
Regressive	10.8%	12.3%	0.975	7.7%	15.4%	0.202
Stationary	10.8%	13.8%		6.2%	18.5%	
Progressive	24.6%	27.7%		26.2%	26.2%	

## Data Availability

No data were available in this study.

## Supplementary Materials

Supplementary Materials Graphical abstract.
