# Supplementary material for: Prognostic Role of Tumor Microenvironment in DLBCL and Relation to Patients' Clinical Outcome: A Clinical and Immunohistochemical Study
Source: Anal Cell Pathol (Amst). 2022 Jan 17;2022:9993496. doi: 10.1155/2022/9993496 (PMC8786528; doi:10.1155/2022/9993496)
Supplement: Supplementary Materials — Graphical abstract. [file 9993496.f1.pdf]

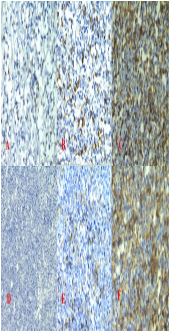

DLBCL SHOWED NO PROGNOSTIC RELATION WITH TUMOR MICROENIVORNMENT

MICROENIVORONMENT MARKERS CD4 CD8 CD 68 MMP1 IMMUNOHISTOCHEMISTRY

Lymphoma Immune stain expression grades
